# The relationship between pancreas steatosis and the risk of metabolic syndrome and insulin resistance in Chinese adolescents with concurrent obesity and non‐alcoholic fatty liver disease

**DOI:** 10.1111/ijpo.12653

**Published:** 2020-04-29

**Authors:** Chileka Chiyanika, Dorothy F. Y. Chan, Steve C. N. Hui, Hung‐kwan So, Min Deng, David K. W. Yeung, E. Anthony S. Nelson, Winnie C. W. Chu

**Affiliations:** ^1^ Department of Imaging and Interventional Radiology The Chinese University of Hong Kong Hong Kong China; ^2^ Department of Paediatrics The Chinese University of Hong Kong Hong Kong China; ^3^ Russell H. Morgan Department of Radiology and Radiological Science The Johns Hopkins University School of Medicine Baltimore Maryland USA; ^4^ Department of Paediatrics and Adolescent Medicine The University of Hong Kong Hong Kong China; ^5^ Department of Clinical Oncology The Chinese University of Hong Kong Hong Kong China

**Keywords:** fatty liver, insulin resistance, magnetic resonance imaging, metabolic syndrome, pancreas

## Abstract

**Background:**

The incidence of childhood obesity and associated comorbidities are on an increasing trend worldwide. More than 340 million children and adolescents aged between 5 and 19 years old were overweight or had obesity in 2016, from which over 124 million children and adolescents (6% of girls and 8% of boys) had obesity.

**Objective:**

To describe the relationship between pancreas steatosis, body fat and the risk of metabolic syndrome, insulin resistance in Hong Kong Chinese adolescents with both obesity and non‐alcoholic fatty liver disease (NAFLD).

**Methods:**

Fifty two adolescents with obesity and NAFLD were analysed (14‐18 years), stratified into fatty and non‐fatty pancreas groups using chemical shift encoded MRI‐pancreas proton density fat fraction ≥5%. Pancreatic, abdominal subcutaneous adipose tissue (SAT)/visceral adipose tissue (VAT) volumes, biochemical and anthropometric parameters were measured. Mann‐Whitney *U* test, multiple linear/binary logistic regression analyses and odds ratios were used.

**Results:**

Fifty percent had fatty pancreas, 38% had metabolic syndrome and 81% had insulin resistance. Liver proton density fat fraction (PDFF) and VAT were independent predictors of insulin resistance (*P* = .006, .016). Pancreas and liver PDFF were both independent predictors of beta cells dysfunction (*P* = .015, .050) and metabolic syndrome (*P* = .021, .041). Presence of fatty pancreas in obesity was associated with insulin resistance (OR = 1.58, 95% CI = 0.39‐6.4) and metabolic syndrome (OR = 1.70, 95% CI = 0.53‐5.5).

**Conclusion:**

A significant causal relationship exists between fatty pancreas, fatty liver, body fat and the risk of developing metabolic syndrome and insulin resistance.

**Key Points:**

- Fatty pancreas is a common finding in adolescents with obesity, with a prevalence rate of 50% in this study cohort.
- Liver PDFF and VAT are independent predictors of insulin resistance while pancreas PDFF and liver PDFF are independent predictors of both beta cells dysfunction and metabolic syndrome.
- Presence of fatty pancreas at imaging should not be considered as a benign finding but rather as an imaging biomarker of emerging pancreatic metabolic and endocrine dysfunction.

**Abbreviations**
BMI: body mass index
HOMA‐B: homeostasis model assessment‐beta
HOMA‐IR: homeostasis model assessment‐insulin resistance
NAFLD: non‐alcoholic fatty liver disease
QUICKI: quantitative insulin sensitivity check index
SAT: subcutaneous adipose tissue
T2DM: type 2 diabetes mellitus
VAT: visceral adipose tissue

## 1 INTRODUCTION

Childhood obesity and its associated comorbidities are increasing.^1^ The excess fat tends to accumulate in undesired areas such as the liver, pancreas, heart, skeletal muscle and visceral adipose tissue.^2^ It is this distribution of fat that plays a critical role in the development of complications^3^ and is understood to pose a risk for insulin resistance.^4^ Lee et al^5^ showed that a third of children and adolescents with obesity have glucose intolerance and relative β‐cell failure. Fatty pancreas for instance has been shown to be a significant risk factor for insulin resistance/diabetes in children and adults.^6^ In adults, fatty pancreas is found to be significantly correlated with β‐cell dysfunction and decreased insulin secretion.^7^

Fatty pancreas has also been associated with metabolic syndrome, which is characterized by central obesity, hypertension, impaired glucose tolerance and dyslipidemia.^8^ Singh et al^9^ showed a 2 fold increased risk of metabolic syndrome in people with fatty pancreas while Elhady et al^6^ showed an increased risk of metabolic syndrome (OR 11.40; CI 95%: 2.69‐48.22) in children with obesity and fatty pancreas, with fatty pancreas being an independent predictor of metabolic syndrome. Maggio et al^1^ demonstrated that increased pancreatic fat was present in adolescents with obesity who also had metabolic syndrome. Likewise, fatty pancreas is further associated with central obesity, which is linked to both insulin resistance and metabolic syndrome.^8^

Interestingly, studies have shown that in people suffering from impaired glucose metabolism there was decreased pancreatic volume and increased pancreatic fat.^10^, ^11^ Other studies also showed that individuals with type 2 diabetes mellitus (T2DM) had a smaller pancreatic volume and higher pancreatic fat when compared to people without T2DM.^12^ Suggesting that a large pancreatic volume may indicate a larger reservoir of beta cells and greater capacity to withstand the various factors that contribute to the development of diabetes.^13^

Chemical shift encoded MRI (CSE‐MRI) is an excellent quantitative method to calculate fat in the body. It is robust, accurate, reproducible, vendor and operator independent method that is able to quantify body, pancreatic and hepatic fat content.^14^ Very limited studies have examined the relationship among fatty pancreas, other ectopic fat deposition areas in the abdomen and the risk of developing metabolic syndrome and insulin resistance in adolescents using magnetic resonance imaging. Most of the available studies used Ultrasound. To the best of our knowledge, to date only four studies involving predominantly European Caucasian,^1^, ^15^, ^16^ African American/Latino^17^ children and adolescents with obesity, with and/or without NAFLD used MRI to evaluate the afore‐mentioned relationship. Based on these findings, the purpose of our study was to utilize CSE‐MRI (mDixon method) to evaluate the relationship of fatty pancreas, whole abdominal subcutaneous/visceral adipose tissues and the risk of developing metabolic syndrome and insulin resistance in Chinese adolescents with both obesity and non‐alcoholic fatty liver disease (NAFLD).

## 2 MATERIALS AND METHODS

### 2.1 Study population

**FIGURE 1:**
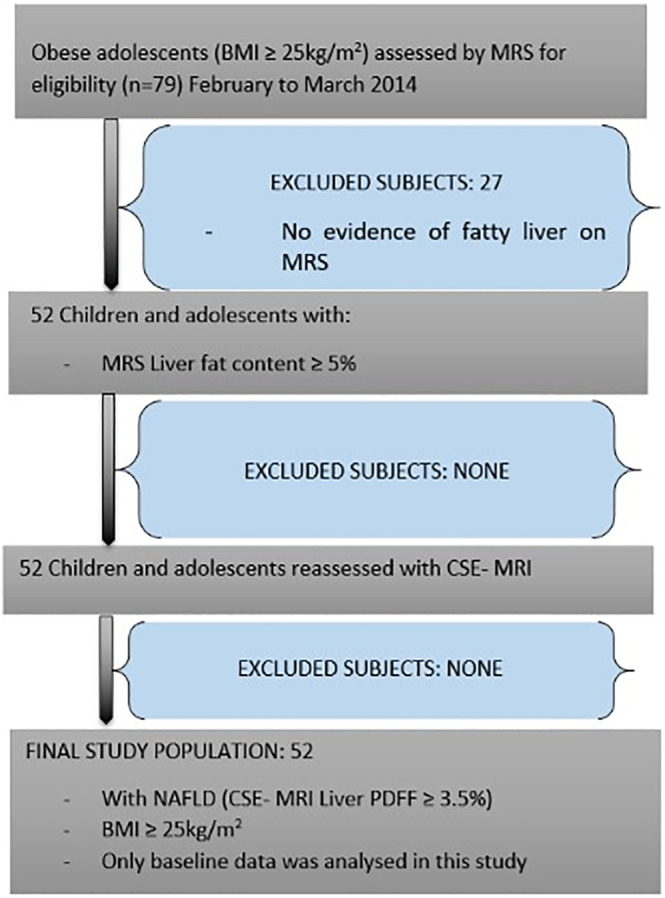
Flow diagram of the study subjects. NAFLD = Non‐alcoholic fatty liver disease, BMI = Body Mass Index, calculated as body weight in kilograms divided by height in metre squared, MRS = Magnetic resonance spectroscopy, PDFF = proton density fat fraction, CSE‐MRI = Chemical shift encoded magnetic resonance imaging

This study was a substudy of Chan et al^18^ reported previously, which evaluated the efficacy of dietitian‐led lifestyle modification programme to reduce non‐alcoholic fatty liver disease (NAFLD) in adolescents with obesity. The study was approved by our institutional review board and written informed consent was obtained from the parents or guardians and all participants assent to participate in the study. Seventy‐nine (79) children with obesity were screened between February 2014 and March 2014 for the presence of liver fat content level of ≥5% by proton magnetic resonance spectroscopy to determine fatty liver.^19^ Fifty two participants were finally enrolled in the study (Figure 1). In order to be more accurate with liver fat measurements, we re‐evaluated the liver fat content in all the 52 participants using chemical shift encoded MRI method. NAFLD was defined as liver proton density fat fraction (PDFF) of ≥3.5% in children and adolescents.^20^ All the participants were found to have NAFLD. Thus, our study cohort included 52 consecutive post‐pubertal Chinese adolescents with both obesity [BMI ≥95th percentile of a local reference^21^] and NAFLD in Tanner stage 5. Exclusion criteria were the history of viral hepatitis, diabetes, alcohol consumption, concurrent participation in another clinical trial, chronic medical illness, metallic implants and other MRI contraindications, and on any treatment with drugs that are known to affect the liver or pancreatic fat.

### 2.2 Physical examinations and anthropometrics

Physical examination and anthropometric measurements were carefully taken at least within 24 hours of performing abdominal MRI scan. These included body weight in kilograms (kg), while the child was in light clothes and bare feet. The height in metres was measured using a flexible non‐stretchable measuring tape with the subject standing upright, extended knees, hips, waist and neck. BMI was calculated as weight (kg) divided by height in metre squared and z‐scores were derived using World Health Organization references.^22^ Obesity was diagnosed if the BMI was ≥95th percentile for age and sex. Waist circumference (WC) was measured at the mid‐point between the lower costal margin and the iliac crest over the unclothed abdomen in the standing position, bare feet, at the end of normal expiration with the measuring tape stretched all around the body in the horizontal position.

### 2.3 Blood pressure (BP)

Participants were allowed to be seated in a quiet room for 3‐5 minutes before measurement to reduce anxiety, with the back supported and feet uncrossed on the floor.^23^ Talking was not allowed during BP measurements. Systolic and diastolic blood pressure was measured from the right arm in all studied children three times, at 2 minutes' intervals using a standard commercially available automated blood pressure machine. The results were recorded as necessary using the lowest reading of the three obtained readings. Hypertension was defined as blood pressure in subjects above 10 years old with systolic and diastolic blood pressure ≥130/85 mm Hg.^24^

### 2.4 Laboratory measurements

Laboratory tests were performed following 8 hours of fasting and within 24 hours of performing an abdominal MRI scan. Investigations included: plasma fasting glucose, lipid profile, plasma alanine aminotransferase, aspartate aminotransferase and serum insulin. Homeostasis model assessment‐insulin resistance (HOMA‐IR), Quantitative insulin sensitivity check index (QUICKI) were used to study insulin resistance while Homeostasis model assessment‐beta (HOMA‐B) was used to study pancreatic beta cells dysfunction. HOMA‐IR was calculated as: Fasting insulin (mIU/L) × glucose (mmol/l)/22.5,^25^ HOMA‐B was calculated as [20 × Fasting insulin (mIU/L)]/[glucose (mmol/l)‐3.5]^26^ while QUICK was calculated as 1/(log fasting glucose (mg/100 mL) + log fasting insulin (mIU/L).^27^

### 2.5 Metabolic syndrome

Metabolic syndrome was defined according to the International Diabetes Federation (IDF) criteria^28^ as follows: Central obesity (waist circumference ≥90th percentile for ages 10‐16 years or waist circumference ≥90 cm (boys) or ≥80 cm (girls) or body mass index (BMI) ≥30 kg/m^2^ for ages above 16 years) plus any two or more: Hypertension (Systolic blood pressure ≥130 mm Hg or Diastolic blood pressure ≥85 mm Hg or treatment with anti‐hypertensive drugs), Dyslipidemia (triglycerides ≥1.7 mmol/L or high density lipoproteins cholesterol levels (HDL‐ch) ≤ 1.0 mmoL/L for ages 10‐16 years or triglycerides ≥1.7 mmoL/L or HDL‐ch ≤1.0 (boys) or ≤1.3 mmoL/L (girls) for ages above 16 years) or impaired glucose (fasting plasma glucose ≥5.6 mmol/L).

### 2.6 MR image acquisition and reconstruction

MR imaging was performed in all subjects using a 3.0 T scanner (Achieva X series, Philips Healthcare, Best, The Netherlands) equipped with 16‐channel SENSE‐XL‐Torso array coil. 3D spoiled chemical‐shift water‐fat mDixon sequence was used (TR = 5.7 ms, first TE/echo spacing = 1.2‐1.4 (ms)/1.0‐1.2 (ms), number of echoes = 6, flip angle = 3°, parallel imaging acceleration = 2, a 15 seconds breath hold technique was employed to acquire co‐registered water, fat, fat‐fraction and T2* image series and was reconstructed with slice thickness/number of slices = 3.0 mm/50. The field of view (FOV) covered the whole abdomen, that is, region from the dome of the diaphragm to the symphysis pubis. Image reconstruction was completed online using Philips mDixon product implementation with the multi‐peak spectral model of fat and T2* correction to increase accuracy and sensitivity.

### 2.7 Image analysis

#### 2.7.1 Pancreas proton density fat fraction (PDFF)

**FIGURE 2:**
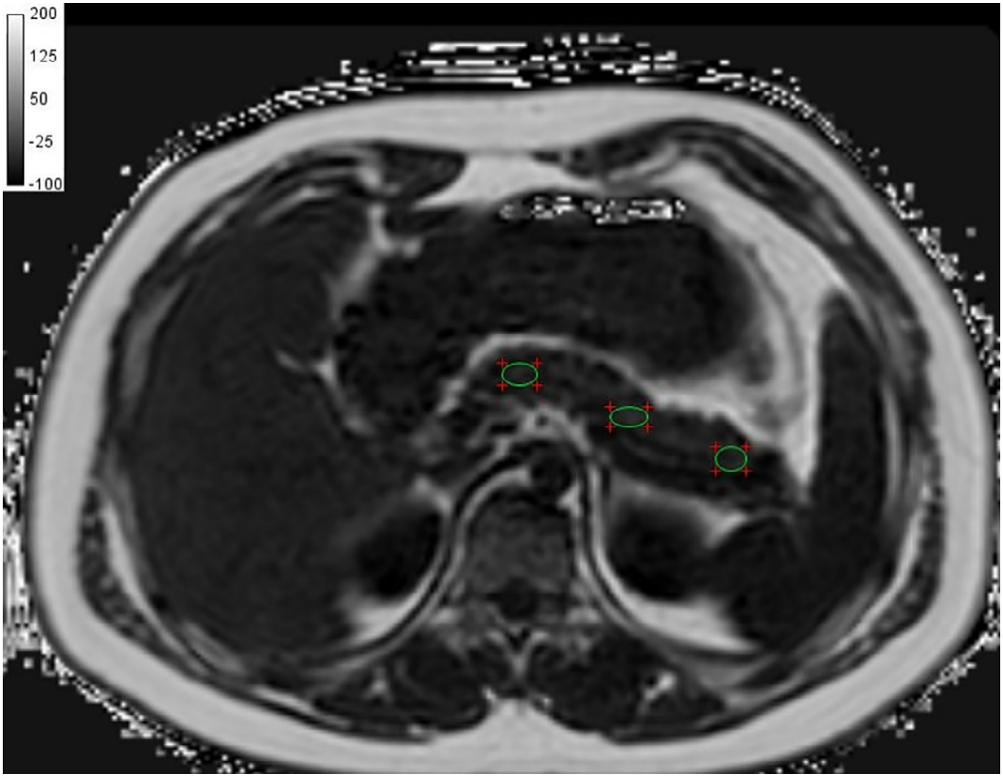
The regions of interest in the pancreas using chemical shift encoded MRI fat fraction image

Readers consisted of two radiology staff with (C.C. PhD Radiology student [Reader 1], D.M. Radiologist [Reader 2]; 15 and 12 years' experience respectively in both abdominal Ultrasound and MR imaging), both of whom were blinded to the clinical data. Pancreas proton density fat fraction (PDFF) was measured using the CSE‐MRI pancreas proton density fat fraction images. Three operator‐defined regions of interest (ROIs) set to 1 cm^2^ were drawn on the head, body and tail of the pancreas thrice in any slice that showed the pancreas clearly (avoiding the pancreatic duct and splenic vein) using RadiAnt DICOM viewer software version 4.6.5, Medixant, Poland as shown in Figure 2. The mean signal intensities from the three ROIs were averaged to get the mean pancreatic fat fraction as the final result. The interclass correlation coefficient was calculated to assess the reliability of the measurements from the two readers. In cases of discrepancies, a consensus was reached by repeating the measurements and then an average was obtained for final analysis. Contrasting to NAFLD, there is no well‐recognized threshold to determine the upper bound of pancreatic fat for healthy individuals or adolescents. However, a study by Maggio et al^1^ recommended that the upper bound normal pancreatic fat fraction in adolescents is 5%, therefore, this cut off was adopted in our study.

#### 2.7.2 Liver proton density fat fraction (PDFF)

Reader 1 determined liver PDFF for each participant using the same RadiaAnt viewer. Nine elliptical regions of interest (ROIs) set to 4 cm^2^ were placed into all nine Couinaud liver segments localized on PDFF maps (obtained in at least two slices) avoiding the hepatic blood vessels, bile ducts and motion artefacts.^29^ The average liver PDFF from all the nine segments was used as the final measurement. All measurements were repeated thrice to define the intraclass correlation coefficient.

### 2.8 Pancreatic volume

**FIGURE 3:**
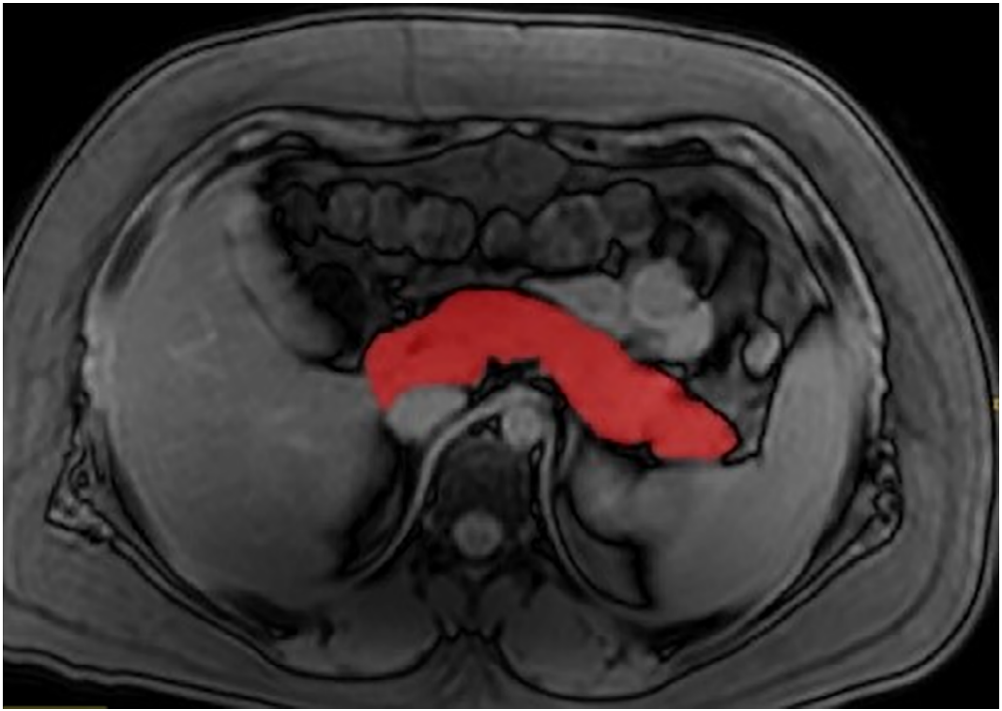
The delineation of the pancreas in a single slice on an out of phase image at the level of the lower border of Lumbar 1 vertebrae

Pancreatic volumes were measured by Reader 1 using CSE‐MRI out of phase images as the pancreatic boundaries are clearest in these series of images. The pancreas was delineated from the adjacent structures using the splenic vein, superior mesenteric vessels, Inferior vena cava, aorta and the duodenum. ITK‐SNAP version 3.6.0^30^ segmentation software was used to delineate the pancreatic tissue in each slice (Figure 3). The pancreatic volumes were measured thrice and the mean volume was used for analysis. These three measurements were also used to define intraclass correlation coefficient.

### 2.9 Abdominal subcutaneous/Visceral adipose tissue

**FIGURE 4:**
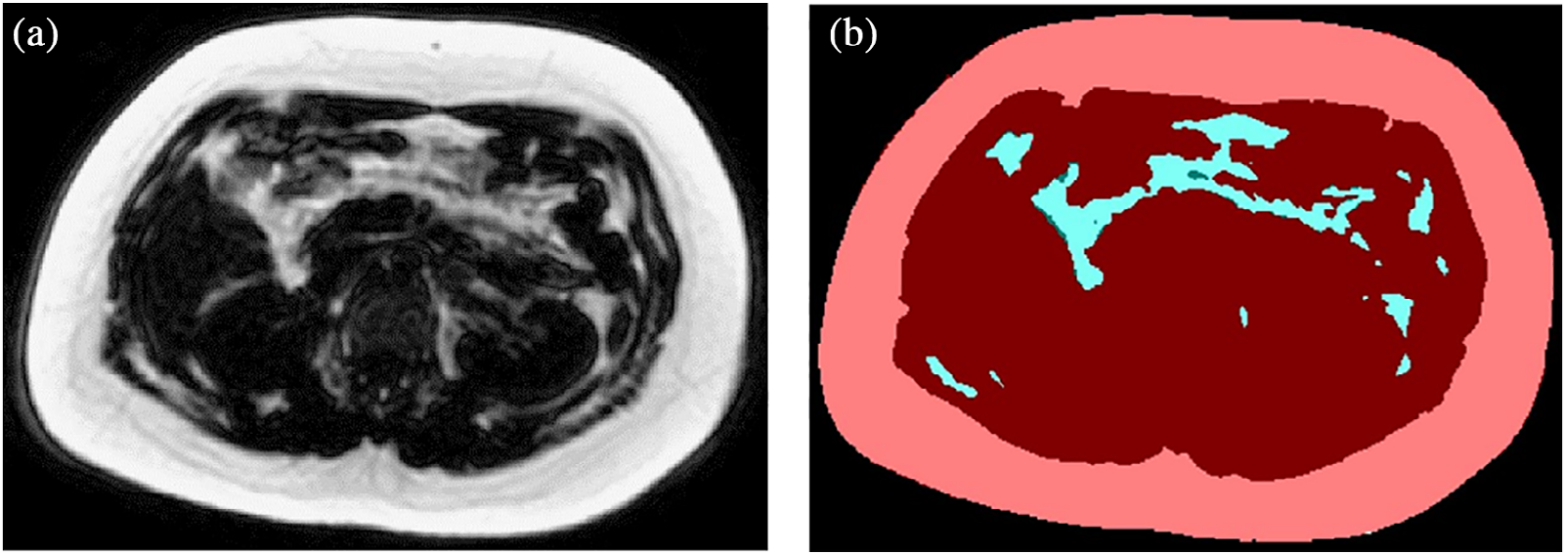
A, The original mDixon MRI fat fraction abdominal image of a single slice obtained at the level of lumbar 3 vertebrae. B, The extracted image from A, showing the subcutaneous adipose tissue (SAT) in colour pink and visceral adipose tissue (VAT) in colour blue using our validated in house method^31^

Using an in‐house automated validated method^31^ developed in MATLAB platform (MATLAB R2011a, MathWorks, Natick, USA), SAT and VAT volumes were extracted from CSE‐MRI proton density fat fraction images of the whole abdomen, that is, region from the dome of the diaphragm to the symphysis pubis as shown in Figure 4. Briefly, this algorithm utilized Bresenham's Line method and Midpoint Circle method to construct a spoke‐like template, and this template was applied to the scan over the adipose tissue to separate SAT and VAT.

### 2.10 Statistical analysis

Normally distributed data was expressed as means ± SD, unless stated otherwise. Differences between two groups were analysed using Mann‐Whitney *U* test. Kruskal‐Wallis test was used to compare the fat distribution in the three regions of the pancreas. Interclass correlation coefficient was used to evaluate the inter and intra reader agreement of ROIs with 1 to 2 weeks' interval between measurements. Pearson's correlation coefficients were used to assess linear relationships between variables. To evaluate the causation relationships between variables, multiple linear and binary Logistic regression analyses with correction for multiple comparisons were used. Relative risk was determined by odds ratios. All tests were two‐sided and P‐values <.05 were considered statistically significant. Statistical analyses were performed by using the SPSS statistical package software (version 25.0; SPSS, Chicago, IL).

## 3 RESULTS

### 3.1 Study population

Fifty‐two participants (15.7 years ±1.2; age range 14‐18 years, BMI z‐score; 2.3 ± 0.4, BMI; 32.4 ± 3.2 kg/m^2^, waist circumference; 103.7 ± 8.7 cm and liver PDFF; 9.8 ± 7.6%) were analysed. The detailed patient characteristics are outlined in Tables 1 and 2. Fifty per cent were diagnosed as having fatty pancreas with interclass correlation coefficient absolute agreement of 0.860 (95% confidence interval [CI]: 0.756, 0.922, *P* < .0001). Participants were categorized into fatty pancreas group (N = 26) and non‐fatty pancreas group (N = 26). Pancreas PDFF and body weight were significantly different between the two groups (*P* < .0001 and *P* = .027). Of note, the fat content was homogenously distributed in all the regions (head, body tail) of the pancreas (*P* < .05). No significant pancreas PDFF differences were noted between sex (*P* = .174).

**TABLE 1:** Comparison of anthropometric and patient characteristics in participants with and without fatty pancreas

Variables	All participants (n = 52)	No fatty pancreas (n = 26)	Fatty pancreas (n = 26)	*P* value
Age (y)	15.7 (1.2)	15.7 (1.1)	15.6 (1.3)	.951
Boys (y)	15.5 (1.1)	15.5 (1.2)	15.5 (1.1)	.942
Girls (y)	16.0 (1.2)	15.8 (1.0)	16.2 (1.5)	.612
Boys, n (%)	33 (63.5)	14 (53.8)	19 (73.1)	.249
Girls, n (%)	19 (36.5)	12 (46.2)	7 (26.9)	1.000
Body weight (kg)	90.9 (8.9)	88.2 (8.2)	93.6 (8.9)	.027^a^
Boys	92.7 (7.6)	91 (6.9)	94 (8)	.433
Girls	87.7 (10.2)	85 (8.7)	92 (11.5)	.091
BMI (kg/m^2^)	32.4 (3.2)	32.1 (3.0)	32.6 (3.4)	.509
Boys	31.7 (3.1)	31.0 (3.1)	32.2 (3)	.071
Girls	33.5 (3.2)	33.1 (2.5)	33.8 (4.3)	.472
BMI z‐score	2.3 (0.4)	2.3 (0.4)	2.3 (0.4)	.780
Boys	2.1 (0.3)	2.1 (0.3)	2.2 (0.3)	.130
Girls	2.6 (0.3)	2.6 (0.2)	2.6 (0.5)	.375
Central obesity (WC) (cm)	103.7 (8.7)	102.8 (6.6)	104.7 (10.4)	.440
Boys	104.9 (8.7)	104.0 (7)	105.6 (9.9)	.610
Girls	101.7 (8.5)	101.3 (6)	102.2 (12.3)	.673
Waist circumference to height ratio	0.62 (0.05)	0.63 (0.05)	0.61 (0.06)	.138
Boys	0.61 (0.06)	0.63(0.06)	0.60(0.06)	.274
Girls	0.63 (0.03)	0.64 (0.03)	0.63(0.03)	.866
WAT volume‐abdomen (L)	16.30 (3.2)	16.0(3.3)	16.7 (3.1)	.417
Boys	15.0 (4.1)	15.0 (3.0)	16.5 (3.1)	.150
Girls	19.5 (5.6)	17.1 (3.3)	17.0 (3.3)	.878
VAT volume‐abdomen (L)	2.9 (0.8)	4.3 (2.9)	4.7 (2.9)	.482
Boys	2.2 (1.1)	2.9 (0.9)	3.0 (0.9)	.647
Girls	3.0 (1.0)	2.7 (1.1)	2.7 (0.8)	.667
SAT volume‐abdomen (L)	13.4 (2.8)	13.1(3.0)	13.8 (2.7)	.390
Boys	12.7 (3.3)	12.1 (2.8)	13.6 ()	.138
Girls	16.5 (4.2)	14.2 (2.8)	14.3 (3.2)	.945
VAT/SAT ratio	0.18(0.18)	0.17(0.07)	0.19(0.08)	.534
Boys	0.17(0.08)	0.17(0.09)	0.18(0.08)	.924
Girls	0.19(0.06)	0.17(0.04)	0.23(0.09)	.269
Pancreatic fat fraction (%)	5.3 (1.7)	4.1 (0.6)	6.5 (1.6)	<.0001^a^
Boys	5.4 (1.5)	4.2 (0.6)	6.3 (1.3)	<.0001^a^
Girls	5.1 (2)	3.9 (0.6)	7.0 (2.1)	<.0001^a^
Pancreatic volume (cm^3^)	73.7 (18.6)	75.1 (20.2)	72.3 (17.1)	.592
Boys	73.3 (21)	76.0 (23.7)	71.5 (19.1)	.749
Girls	74.3 (14.5)	74.3 (16.6)	74.2 (11.1)	1.000
Liver proton density fat fraction (%)	9.8 (7.6)	10.3 (8.7)	9.3 (6.6)	.927
Boys	9.2 (6.8)	8.7 (6.2)	9.5 (7.4)	.942
Girls	10.9 (9.0)	12.0 (10.9)	8.9 (4.1)	.800
Systolic blood pressure (mm Hg)	128 (17)	126 (19)	131 (14)	.280
Diastolic blood pressure (mm Hg)	71 (11)	70 (10)	72 (11)	.484
Hypertension n (%)	24 (46.2)	9(34.6)	15 (57.7)	.082

*Note*: Values are mean (SD) or numbers (percentages).

Abbreviations: ALT, alanine aminotransferase; AST, aspartate aminotransferase; BMI, body mass index; HDL, high‐density lipoprotein; HOMA‐B, homoeostasis model assessment‐beta cell function; HOMA‐IR, homoeostasis model assessment‐Insulin resistance; LDL, low‐density lipoprotein; MRS, magnetic resonance spectroscopy; QUICKI, quantitative insulin‐sensitivity check index; SAT, subcutaneous adipose tissue; VAT, visceral adipose tissue; WAT, white adipose tissue; WC, waist circumference‐a measure of central obesity.

a Indicates significant difference using Mann–Whitney *U* test.

**TABLE 2:** Comparison of biochemical markers in participants with and without fatty pancreas

Variables	All participants (n = 52)	No fatty pancreas (n = 26)	Fatty pancreas (n = 26)	*P* value
ALT (IU/L)	37.3 (25)	36.7 (21.3)	37.8 (26.7)	.877
AST (IU/L)	22.7 (8.4)	22.7 (7.9)	22.6 (9.1)	.834
AST/ALT ratio	0.73 (0.3)	0.72 (0.2)	0.74 (0.3)	.821
Serum insulin (mIU/L)	27.6 (19.8)	24.1 (13.0)	31.1 (24.6)	.210
Fasting plasma glucose (mmol/L)	4.9 (0.4)	4.9 (0.4)	4.9 (0.5)	.689
HOMA‐IR	5.8 (3.7)	5.4 (3.2)	6.2 (4.1)	.440
HOMA‐B	55.36 (19.3)	56.3 (21.5)	54.3 (16.7)	.671
QUICKI	0.5 (0.1)	0.5 (0.1)	0.5 (0.1)	.274
Total cholesterol (mmol/L)	4.1(0.8)	4.1 (0.7)	4.1 (0.8)	.970
HDL‐cholesterol (mmol/L)	1.2 (0.2)	1.1(0.3)	1.2(0.2)	.267
LDL‐cholesterol (mmol/L)	2.4 (0.7)	2.4 (0.8)	2.4 (0.8)	.897
Triglycerides (mmol/L)	1.1 (0.4)	1.1(0.4)	1.0 (0.4)	.460
Metabolic syndrome n (%)	17 (32.7)	7(26.9)	10 (38.5)	.278
Insulin resistance‐HOMA‐IR n (%)	41 (78.8)	20 (76.9)	21(80.8)	.390

*Note*: Values are mean (SD) or numbers (percentages).

Abbreviations: ALT, alanine aminotransferase; AST, aspartate aminotransferase; BMI, body mass index; HDL, high‐density lipoprotein; HOMA‐B, homoeostasis model assessment‐beta cell function; HOMA‐IR, homoeostasis model assessment‐Insulin resistance; LDL, low‐density lipoprotein; QUICKI, quantitative insulin‐sensitivity check index.

### 3.2 Blood biochemistry analysis

No statistically significant differences were observed between groups in all blood biochemical markers, lipid profiles, HOMA‐IR and HOMA‐B. However, there was a trend of higher HOMA‐IR, serum insulin and lower HOMA‐B (higher beta cells dysfunction) in the fatty pancreas group. 79% of all the participants were found to have insulin resistance, that is, HOMA‐IR ≥2.6^32^ but all had normal QUICKI ≥0.36.^33^ The proportion of participants with insulin resistance was not different between the two groups, that is, 77% vs 81% *P* = .390, in the non‐fatty pancreas group vs fatty pancreas group respectively. Metabolic syndrome was diagnosed in 27% vs 39% of the participants in the non‐fatty pancreas group vs fatty pancreas group respectively, *P* = .38. Hypertension was diagnosed in 35% vs 58% of the participants in the non‐fatty pancreas group vs fatty pancreas group, *P* = .098.

### 3.3 Radiological analysis

The mean liver PDFF between groups was not statistically different (10.3% vs 9.3%, *P* = .927) non‐fatty pancreas vs fatty pancreas respectively with intraclass correlation coefficient absolute agreement of 0.936 (95% confidence interval [CI]: 0.805, 0.980, *P* < .0001). To assess the relationship between pancreatic volume and insulin resistance/beta cells dysfunction using HOMA‐IR/ HOMA‐B, the pancreatic volumes were calculated. The mean pancreatic volume of the whole study population was 73.7± 18.6 cm^3^. No statistically significant differences were noted between the groups (*P* = .592), with intraclass correlation coefficient absolute agreement of 0.908 (95% confidence interval [CI]: 0.791, 0.961, *P* < .0001).

### 3.4 The relationship between anthropometric, body fat, glycemic biochemical parameters, fatty liver and fatty pancreas

Pearson correlation coefficient tests showed that liver PDFF correlated with central obesity, BMI, Homeostasis model assessment‐insulin resistance (HOMA‐IR), Homeostasis model assessment‐beta (HOMA‐B), triglycerides, SAT and metabolic syndrome (*P* = .017, .029, .010, .026, .011, .028, .015). Pancreas PDFF correlated with body weight, BMI and central obesity (*P* = .002, .012, .030), with a borderline correlation with HOMA‐IR (*P* = .056). SAT correlated with body weight, BMI, BMI z‐score, central obesity, HOMA‐IR and HOMA‐B (*P* < .001, <.001, <.001 < .001, .048, .019). VAT correlated with age, BMI, BMI z‐score, central obesity, triglycerides and HOMA‐IR (*P* = .031, .042, .035, .031, .002, .003). Summary of the correlation results are shown in Table 3.

**TABLE 3:** Correlations between anthropometric, glycemic biochemical parameters, fatty liver, fatty pancreas and body fat

	Liver fat content		Pancreatic fat content		VAT		SAT	
	r	*P* value	r	*P* value	r	*P* value	r	*P* value
Age (y)	−0.128	.366	−0.221	.115	0.302*	.031	−0.083	.561
Body weight (kg)	0.181	.202	0.425**	.002	0.264	.061	0.528**	.000
BMI	0.302*	.029	0.346*	.012	0.286*	.042	0.675**	.000
BMI z‐score	0.262	.061	0.193	.171	0.296*	.035	0.717**	.000
Central obesity (cm)	0.331*	.017	0.301*	.030	0.299*	.031	0.671**	.000
SAT (cm^3^)	0.347*	.028	0.129	.366	‐	‐	‐	‐
VAT (cm^3^)	0.169	.235	0.123	.390	‐	‐	‐	‐
Serum insulin (mIU/L)	0.211	.132	0.236	.092	0.200	.159	0.201	.157
Plasma fasting glucose (mmol/L)	0.095	.501	−0.203	.149	0.107	.457	0.053	.712
Triglycerides (mmol/L)	0.338*	.011	0.137	.331	0.417*	.002	0.194	.173
HOMA‐IR	0.358*	.010	0.269	.056	0.408*	.003	0.278*	.048
HOMA‐B	0.308*	.026	0.258	.065	0.327	.094	0.328*	.019
Metabolic syndrome	0.335*	.015	0.264	.059	0.086	.549	−0.079	.582
Pancreatic volume (cm^3^)	0.180	.202	−0.108	.462	0.043	.768	−0.070	.631
Pancreatic fat	0.144	.310	‐	‐	‐	‐	‐	‐

Abbreviations: BMI, body mass index; HOMA‐B, homoeostasis model assessment‐Insulin resistance; HOMA‐B, homoeostasis model assessment‐beta cell function; SAT, subcutaneous adipose tissue; VAT, visceral adipose tissue.

* Pearson correlation significant at the *P* < .05 level (2 tailed).

** Pearson correlation significant at the *P* < .01 level (2 tailed).

### 3.5 Regression and relative risks analyses

Multiple linear regression step‐wise analysis showed that BMI (B = 0.284, *P* < .001), total cholesterol (B = 0.808, *P* = .009) and plasma fasting glucose (B = ‐1.079, *P* = .034) were independent predictors of fatty pancreas after correcting for age, sex, HOMA‐IR, triglycerides, SAT, VAT, LDL‐c, HDL‐c, insulin and central obesity. Multiple linear regression step‐wise analysis showed that VAT and liver PDFF (B = 0.001, *P* = .006 and B = 0.130, *P* = .016, respectively) were the only independent predictors of insulin resistance after correcting for pancreas PDFF, SAT, BMI and pancreatic volume.

Pancreas PDFF (B = 103.63, *P* = .015) and liver PDFF (B = 17.14, *P* = .050) were independent predictors of beta cells dysfunction at multiple regression enter method after correcting for age, VAT, SAT, BMI z‐score and BMI. Binary logistic regression showed that liver and pancreas PDFF (B = 0.109, *P* = .021 and B = 0.500, *P* = .041 respectively) were the independent predictors of metabolic syndrome after correcting for VAT, SAT, BMI and central obesity. In order to know the relative risks of developing insulin resistance and metabolic syndrome given that one is a Chinese adolescent with obesity and has both fatty pancreas and fatty liver, we calculated the odds ratios. It was shown that insulin resistance was associated with fatty pancreas with an odds ratio (OR) 1.575 (95% confidence interval [CI]: 0.39, 6.4) while metabolic syndrome was associated with fatty pancreas with an odds ratio (OR) 1.696 (95% confidence interval [CI]: 0.53, 5.5).

## 4 DISCUSSION

Excess body fat tends to accumulate in ectopic areas, and is associated with metabolic diseases.^34^ Unlike the liver that has been widely studied in relation to obesity related comorbidities, limited studies utilizing MRI are available that demonstrated the relationship among fatty pancreas, body fat and the risk of metabolic syndrome and insulin resistance in adolescents with both obesity and non‐alcoholic fatty liver disease. In this study it has been demonstrated that fatty pancreas is a common finding (50%) among Chinese adolescents with concurrent obesity and NAFLD. Fatty pancreas, fatty liver and visceral adipose tissue (VAT) were shown to be interrelated, mediated by general and central obesity and were significant risk factors in the development of insulin resistance and metabolic syndrome.

The prevalence of fatty pancreas in this present study is in agreement with the literatures range between 44% and 58%^6^, ^33^, ^35^ in both adolescent and adult cohorts. The discrepancies in the prevalence rates could be attributed to the radiological modality used for the diagnosis of fatty pancreas as well as the ethnicity of the study groups. Unlike our study that utilized CSE‐MRI method, the other studies used ultrasound for diagnosis of fatty pancreas based on sonographic echogenicity. On ethnicity, Lê et al^36^ showed that pancreatic fat accumulation varies in different ethnicities. To our knowledge, there is no report about incidence of fatty pancreas in Chinese adolescents.

This study showed that the independent predictors of fatty pancreas are BMI, fasting plasma glucose and total cholesterol, in agreement with a previous study.^37^ Of note, BMI as the highly significant independent factor (over glucose and total cholesterol) to the development of fatty pancreas could be another probable explanation why the prevalence rate of fatty pancreas was 50% in our study, especially that the definition of obesity in the Chinese population uses lower BMI (Kg/m^2^) cut offs.^38^, ^39^ It was also shown that both pancreas and liver PDFF were independent predictors of beta cells dysfunction. Additionally, liver PDFF showed a significant linear association with HOMA‐B in agreement with a previous study.^40^ Tushuizen et al^41^ and Van der Zijl et al^42^ have shown that fatty infiltration of the pancreas contributes to β‐cell dysfunction and possibly to the subsequent development of type 2 diabetes in susceptible humans. Utzschneider et al^40^ demonstrated that subjects with increased liver PDFF had lower systemic insulin sensitivity and decreased β‐cells function. What has been observed in the current study and other similar studies suggest the potential role of both pancreas and liver PDFF in the genesis of β‐cells dysfunction, though this outcome needs be ascertained and validated by future longitudinal study.

This study showed that the odds ratio of developing insulin resistance in adolescents with both obesity and fatty pancreas was nearly 2 folds than in those without fatty pancreas, similar to the findings of Singh et al^43^ while Elhady et al^6^ found an odds ratio of nearly eight. Interestingly, a multiple linear regression analysis showed that liver PDFF and VAT were independent predictors of insulin resistance, similar to other studies.^15^, ^44^ Furthermore, Liver PDFF and VAT correlated with HOMA‐IR in agreement with previous studies^45^, ^46^ while pancreas PDFF had a non‐significant (borderline, *P* = .056) correlation with HOMA‐IR in concordance with previous studies.^10^, ^47^ This implies that increased liver PDFF and VAT are strongly linked to insulin resistance than pancreas PDFF, especially in subjects with concurrent obesity and NAFLD as in our study. Based on these findings, we can postulate that excess liver fat has a more active role than that of excess pancreatic fat in the genesis of IR vis‐à‐vis beta cells dysfunction. Thus, inhibiting the effects of excess pancreatic fat. Furthermore, the portal‐visceral hypothesis consolidates this argument, which states that increased VAT has increased lipolytic activity resulting in increased delivery of free fatty acids and inflammatory cytokines directly to the liver^48^ through the portal system (via Randle's effect) and ultimately leading to insulin resistance.^49^ Accordingly, the mechanism underlying ectopic fat distribution in the liver and pancreas with resultant insulin resistance may be different. Therefore, these results suggest that increased liver PDFF and VAT play a primary and critical role in the development of insulin resistance vis‐à‐vis type 2 diabetes mellitus (T2DM) while increased pancreas PDFF plays an additional role. However, despite the finding that the role of fatty pancreas in the development of insulin resistance is an adjunct one, its presence appears to indicate a “worsening metabolic condition” in an individual. As opposed to our findings, other studies have shown that increased pancreatic fat plays a primary role in the development of insulin resistance vis‐à‐vis T2DM.^6^, ^7^, ^15^, ^50^, ^51^, ^52^

Binary logistic regression showed that both pancreas and liver PDFF were independent predictors of metabolic syndrome similar to other studies.^6^, ^53^ We further showed that the odds ratio of metabolic syndrome in adolescents with obesity and fatty pancreas was 2 folds than in those without fatty pancreas, similar to findings of Singh et al.^9^ These findings support the hypothesis that fatty pancreas and fatty liver are a part of the metabolic syndrome. They further reiterate the essential role of pancreas and liver in glucose and energy homeostasis/lipid metabolism. Any disruption of the anatomical integrity such as ectopic fat infiltration has the potential to distort organ function resulting in metabolic disorders and associated complications such as T2DM and cardiovascular diseases. Therefore, in our opinion, the presence of fatty pancreas at imaging should not be considered as a benign finding but rather as an imaging biomarker of pancreatic metabolic and endocrine dysfunction. This could serve as a wake‐up call to the clinicians to prioritize these patients under early interventional programme with best possible treatment option to reverse the vulnerable metabolic situation.

Strangely, no direct correlations among pancreas PDFF, liver PDFF and VAT were noted in our study similar to the findings of van der Zijl et al.^42^ However, BMI and central obesity correlated significantly with all these parameters including SAT in agreement with a previous study.^51^ These findings imply that the relationship that exists among these parameters is mediated by both general and central obesity. In view of the above inter‐related associations and correlations, we postulate that any early intervention aimed at reducing excess body fat would have a ripple effect in reducing ectopic fat within the liver, pancreas and other visceral organs like kidneys, resulting in reduced risks of metabolic syndrome, insulin resistance and possibly minimize long term complications such as T2DM.

Thus, we would recommend “screening” in this population group once the waist circumference and BMI/BMI z‐scores are outside the normal range. Furthermore, as total cholesterol and plasma fasting glucose were found to be independent predictors of fatty pancreas, we would recommend initiating “screening” if these parameters are elevated. Moreover, as the presence of fatty pancreas was as high as 50% in this Hong Kong Chinese cohort with fatty liver, the presence of fatty liver could be an indicator of the presence of fatty pancreas. Since fatty liver and fatty pancreas can be assessed during the same scanning session using chemical shift encoded MRI (CSE‐MRI) PDFF, the assessment of pancreatic fat can be performed in addition to assessment of hepatic fat.

For limitation, this study only involved a cohort of adolescents with both obesity and NAFLD without healthy controls. Insulin resistance and beta cells function were not directly measured. Instead we used HOMA‐IR and HOMA‐B, acceptable surrogates to measure insulin resistance and beta cells function respectively. Besides, a relatively small sample size may also reduce the statistical power of our measurements. Finally, our study cohort was predominantly Chinese, therefore caution should be taken in the generalization of the results.

In the future, it will be valuable to provide longitudinal follow up for those participants with high risk of developing insulin resistance and metabolic syndrome into their adulthood, to monitor their biochemical profile. With our established quantitative imaging and analysis technique for fat content in abdominal viscera, it would also be interesting to evaluate the change of pancreas. PDFF, liver PDFF and body fat components in participants who might be undergoing life‐modifying intervention or other kinds of medical treatment, to correlate the changes of body fat content with metabolic risks. Additionally, Wong et al^54^ showed the utility of pancreatic PDFF in an adult population study in Hong Kong. As the procedure is simple and straightforward, such a study can be extended to study groups with individuals who do not have obesity and/or without NAFLD.

In conclusion, fatty pancreas is a common finding in Chinese adolescents with both obesity and non‐alcoholic fatty liver disease. A significant causal relationship exists between fatty pancreas, fatty liver, body fat and the risk of developing metabolic syndrome and insulin resistance.

## 5 CONFLICT OF INTEREST

The authors declare that they have no conflict of interest.

## AUTHORS CONTRIBUTIONS

C.C. and W.C. conceptualized the study. S.H. and D.Y. carried out the technical aspect of the experiments. C.C., D.C., H.K.S., M.D. and T.N. were involved in investigation and analysis. D.C. and W.C. sourced the project funding. C.C. wrote the initial draft of the manuscript. All authors revised the manuscript, approved the final manuscript as submitted, and agreed to be accountable for all aspects of the work.
